# Completeness and accuracy of crash outcome data in a cohort of cyclists: a validation study

**DOI:** 10.1186/1471-2458-13-420

**Published:** 2013-05-01

**Authors:** Sandar Tin Tin, Alistair Woodward, Shanthi Ameratunga

**Affiliations:** 1 Section of Epidemiology and Biostatistics, School of Population Health, University of Auckland, Auckland, New Zealand

**Keywords:** Bicycling, Wounds and injuries, Validation studies, Capture recapture, Medical record linkage, Self-report

## Abstract

**Background:**

Bicycling, despite its health and other benefits, raises safety concerns for many people. However, reliable information on bicycle crash injury is scarce as current statistics rely on a single official database of limited quality. This paper evaluated the completeness and accuracy of crash data collected from multiple sources in a prospective cohort study involving cyclists.

**Methods:**

The study recruited 2438 adult cyclists from New Zealand’s largest mass cycling event in November 2006 and another 190 in 2008, and obtained data regarding bicycle crashes that were attended by medical personnel or the police and occurred between the date of recruitment and 30 June 2011, through linkage to insurance claims, hospital discharges, mortality records and police reports. The quality of the linked data was assessed by capture-recapture methods and by comparison with self-reported injury data collected in a follow-up survey.

**Results:**

Of the 2590 cyclists who were resident in New Zealand at recruitment, 855 experienced 1336 crashes, of which 755 occurred on public roads and 120 involved a collision with a motor vehicle, during a median follow-up of 4.6 years. Log-linear models estimated that the linked data were 73.7% (95% CI: 68.0%-78.7%) complete with negligible differences between on- and off-road crashes. The data were 83.3% (95% CI: 78.9%-87.6%) complete for collisions. Agreement with the self-reported data was moderate (kappa: 0.55) and varied by personal factors, cycling exposure and confidence in recalling crash events. If self-reports were considered as the gold standard, the linked data had 63.1% sensitivity and 93.5% specificity for all crashes and 40.0% sensitivity and 99.9% specificity for collisions.

**Conclusions:**

Routinely collected databases substantially underestimate the frequency of bicycle crashes. Self-reported crash data are also incomplete and inconsistent. It is necessary to improve the quality of individual data sources as well as record linkage techniques so that all available data sources can be used reliably.

## Background

Regular cycling provides health and other benefits [1-4]. However, in New Zealand, using a bicycle is not an attractive mode of travel for many people [5] and accounts for only 2% of total travel time [6]. Cycling is becoming more popular as a sport but just over one-fifth of adults reported participating in either road cycling or mountain biking at least once over twelve months in a recent national survey [7].

For many people, safety concerns are one of the major barriers to riding a bicycle [8,9]. For each million hours that were spent cycling on New Zealand roads, according to the official statistics, 29 deaths or injuries resulted from collisions with a motor vehicle [10] and 31 injuries resulted in death or hospital inpatient treatment [11]. Furthermore, almost as many bicycle crashes occurred off-road [12].

However, current statistics typically refer to a single official data source, most commonly police crash reports and less frequently hospital records. These data sources are known to disproportionately undercount bicycle crashes [13-15]. This is not surprising as many bicycle crashes do not come to the attention of the police or medical personnel, and this undercount amounted to 70% or more of self-reported crashes in overseas studies [16,17]. While self-reports can provide information on unreported crashes, their validity may be questionable also, for example, due to nonresponse [18], failure to recall [19] and the influence of socially desirable responses [20]. For all these reasons, it has been proposed that “unattended” bicycle crash injuries are excluded when developing indicators of injury incidence [21].

Even for the crashes that were attended medically or by the police, routinely collected databases may not be complete [13,15] and accurate [22]. Moreover, as the crash data are usually collected for specific administrative purposes, each data source typically captures only a fraction of all crashes [14]. Therefore, using multiple data sources through record linkage may provide a broader, more complete and truer picture of injury, at a relatively low cost.

This paper evaluated the completeness and accuracy of bicycle crash data collected by self-report and by record linkage drawing on four national, routinely collected databases.

## Methods

### Design, setting and participants

The Taupo Bicycle Study is a prospective cohort study of cyclists designed to examine factors associated with regular cycling and injury risk. The sampling frame comprised cyclists, aged 16 year and over, who enrolled online in the Lake Taupo Cycle Challenge. This is New Zealand’s largest mass cycling event, which is held each November and attracts about 10000 cyclists. Participants have varying degrees of cycling experience and they range from competitive sports cyclists and experienced social riders to relative novices of all ages.

Recruitment was undertaken at the time of the 2006 event for the majority of participants, as described, in detail, elsewhere [23]. In brief, email invitations, containing a hyperlink to an information page describing the study, were sent to 5653 participants who provided their email addresses at registration for the event. Those who agreed to take part in the study were taken to a page containing a web questionnaire and asked about demographic characteristics, general cycling activity, previous crash experience and use of injury preventive measures. The questionnaire was completed and submitted by 2438 cyclists (43.1% response rate). Another 190 cyclists were recruited from the 2008 event by including a short description about the study in the event newsletter. Ethical approval was obtained from the University of Auckland Human Participants’ Ethics Committee.

### Crash outcome data

Crash outcome data were collected through record linkage to insurance claims, hospital discharges, mortality records and police reports, covering the period from the date of recruitment to 30 June 2011. Record linkage was undertaken by the data custodians using name, gender, date of birth and address as identifiers. All participants consented to link their data to these databases. In addition, a follow-up survey was conducted in December 2009.

#### Insurance claims

In New Zealand, the Accident Compensation Corporation (ACC) provides personal injury cover for all residents and temporary visitors to New Zealand no matter who is at fault. The claims database is a major source of information on relatively minor injuries with over 80% of the claims related to primary care (e.g., GPs, emergency room treatment) only [24].

Approval for record linkage was obtained from the ACC Research Ethics Committee. A probabilistic linkage followed by a clerical review was undertaken and all claims for bicycle crashes were extracted. The data extracted contain nature and mechanism of injury, health service utilisation and out of hospital cost. Crashes that occurred on public roads and crashes that involved a collision with a motor vehicle were identified from relevant variables as well as from the free text field describing the crash.

#### Hospital discharge and mortality data

These databases are maintained by the Ministry of Health’s Information Directorate. The National Minimum Dataset (NMDS) contains information about inpatients and day patients discharged after a minimum stay of three hours from all public hospitals and over 90% of private hospitals in New Zealand [25,26]. The Mortality Collection contains information about all deaths registered in the country [27].

Participant data were matched to a National Health Index (NHI) number, a unique identifier assigned to every person who uses health and disability support services in New Zealand. An electronic match was made where possible, followed by two stages of manual matching for participants who could not be linked electronically. Of 2590 participants who were resident in New Zealand at recruitment, 99.0% were successfully matched. All hospital discharges and deaths due to injuries or other health conditions were extracted.

The hospital discharge data contain diagnoses and diagnostic and therapeutic procedures undertaken in each hospital visit, which are coded under ICD-10-AM. Cycle crashes were identified using the E codes V10-V19; those that occurred on public roads were identified using the E codes V10-V18.3-9, V19.4-6, V19.9; and those that involved a collision with a motor vehicle were identified using the E codes V12-V14, V19.0-2 and V19.4-6. Readmissions were identified as described previously [28] and excluded.

The mortality data contain the underlying cause of death which is coded under ICD-10-AM and is also described in free text fields. However, the coroners’ reports on the cause of injury death were available only up to 31 December 2008. All deaths due to a bicycle crash were identified from the available data.

#### Police reports

In New Zealand, it is mandatory that any fatal or injury crash involving a collision with a motor vehicle on a public road be reported to the police. A Traffic Crash Report is then completed and sent to the New Zealand Transport Agency where the data are entered in to the Crash Analysis System (CAS) database.

A deterministic linkage followed by a clerical review was undertaken and all bicycle collisions were extracted. The linked data contain location, time and circumstances of the crash, and severity of injury.

#### Follow-up survey

The survey was conducted in December 2009 using a web questionnaire. The questions asked included: the total number of bicycle crashes experienced during the preceding year, the number of crashes for which claims were lodged with ACC, the number of crashes requiring hospital admission, and the number of crashes that were reported to the police. The participants were also asked to indicate the degree of confidence they had regarding the accuracy of their answers to each question using a five-point scale (very unsure, quite unsure, about 50/50, quite sure, very sure). This confidence rating has been shown to be a useful indicator of recall accuracy for physical activity measures [29].

A total of 1537 participants (58.5%) completed the questionnaire, of whom 70 reported not cycling in the preceding year.

### Analyses

A capture-recapture analysis was undertaken to estimate the number of crashes that had occurred which were not identified through record linkage. In addition, the linked data were compared with the self-reported data collected in the follow-up survey.

#### Capture- recapture analysis

Capture-recapture methods were originally developed to estimate the size of an animal population, based on proportions of animals that were captured, marked, released and recaptured in two or more random samples. The procedure assumed closeness of the population, mark integrity, independence of the samples and equal probability of being captured in each sample [30]. Since then, similar methods have been applied in epidemiological studies [31].

For this analysis, the study sample was restricted to the 2590 participants who were resident in New Zealand at recruitment. For each participant, bicycle crashes identified from the different databases were matched based on the date of crash allowing for a two-day difference. Log-linear models were used to estimate missing crashes, taking into account possible associations across the databases. The models were fitted to the incomplete multiway contingency table with one missing cell corresponding to absence in all databases. The strength of evidence for each model was assessed using Akaike’s Information Criterion (AIC) and its weight. Based on the model averaged estimate and unconditional standard error, the frequency for the missing cell and its 95% confidence interval (CI) were calculated. Analyses were undertaken for bicycle crashes in general, and also for the specific categories of on-road crashes and crashes involving a collision with a motor vehicle.

#### Comparison with self-reports

This analysis was based on the 1456 participants who completed the follow-up questionnaire and reported cycling in the preceding year. As some participants may have experienced more than one crash during the specified period, the exact crash date was not asked in the questionnaire. As such, it was not possible to match the linked and self-reported data for each crash identified in the source databases. Instead, agreement was assessed on a person-to-person basis for each database as well as for the combined data. Agreement was established (1) if a participant reported at least one bicycle crash that required medical attention (that is, involved a claim lodged with ACC or required an admission to hospital) or reported to the police in the preceding year, and the linked data also showed at least one bicycle crash during the same period, or (2) if such a crash had not been experienced in the preceding year according to both the self-reported and linked data.

Cohen’s kappa coefficients were used to determine the degree of agreement. In addition, the sensitivity, specificity and predictive values of the linked data were calculated, assuming that self-reports were the gold standard. Analyses were undertaken for all crashes as well as those involving a collision with a motor vehicle. In addition, subgroup analyses were performed for all crashes to examine differences in agreement by participants’ demographic characteristics, amount of cycling, pre-existing medical conditions (heart attack, stroke, cancer, diabetes or high blood pressure) and confidence in recall.

SAS 9.2 (SAS Institute, Cary, North Carolina) and Microsoft Office Excel 2010 (Microsoft Corporation, Redmond, Washington) were used for all analyses.

## Results

The average age of the participants was 44.0 years (SD 10.4) and 72.4% were males (Table 1). About half the sample were university graduates (53.9%) and lived in least deprived neighbourhoods (49.9%), and 77.7% lived in main urban areas. On average, participants cycled 5.7 hours a week (SD 3.7; Quartile Range 5).

**Table 1:** Participants’ demographic characteristics

**Baseline Characteristics**	**N**	**%**
***Total***	**2590**	
***Age (years)***		
16-35	579	22.4
36-50	1351	52.2
51+	660	25.5
***Gender***		
Male	1874	72.4
Female	715	27.6
***Ethnicity***		
Māori	104	4.0
Non-Māori	2486	96.0
***Level of education***		
High school (secondary) or less	535	20.7
Polytechnic	654	25.3
University	1395	53.9
Missing	6	0.2
NZDep2006 scores*		
1-3	1292	49.9
4-7	919	35.5
8-10	343	13.2
Missing	36	1.4
***Urbanity of residence***		
Main urban area	2013	77.7
Others	541	20.9
Missing	36	1.4
***Region of residence***		
Auckland	919	35.5
Wellington	534	20.6
Others	1101	42.5
Missing	36	1.4

* 2006 New Zealand Deprivation Index with decile ten the most deprived neighbourhood and decile one the least.

### Bicycle crashes reported at the follow-up survey

Of the 1456 participants who completed the follow-up questionnaire and reported cycling in the preceding year, 432 reported experiencing one or more crashes in the preceding year (Table 2). There were a total of 784 self-reported crashes, of which 57.4% occurred on the road and 17.9% involved a collision with a motor vehicle. Based on the respondent reports, 29.1% of all crashes involved a claim lodged with ACC, 3.7% required hospital admission and 6.5% were reported to the police. A higher proportion of collisions involved medical or police attention with 35.0% resulting in claims to ACC, 7.1% requiring hospital admission and 32.9% being reported to the police.

**Table 2:** Bicycle crashes reported by participants at the follow-up survey

	**No. of crashes (No. of participants)**		
	**All crashes**	**On-road crashes**	**Collisions**
Total crashes experienced in the preceding year	784 (432)	450 (324)	140 (106)
Crashes claimed to ACC	228 (173)		49 (41)
Crashes requiring hospital admission	29 (29)		10 (10)
Crashes reported to police	51 (48)		46 (44)

### Bicycle crashes identified through record linkage

During a median follow-up of 4.6 years, only one death occurred due to a bicycle crash. As this fatal crash was recorded in both the Mortality Collection and NMDS databases, the former was excluded in further analysis.

Of the 2590 participants, 855 experienced 1336 bicycle crashes recorded in one or more databases, of which 755 (56.5%) occurred on public roads and 120 (9.0%) involved a collision with a motor vehicle. Only 18 crashes that involved a collision with a motor vehicle were identified from all databases (Table 3).

**Table 3:** Bicycle crashes matched across different data sources

**ACC**	**NMDS**	**CAS**	**No. of crashes**	**% of total crashes**
***All crashes***				
1	1	1	18	1.3
0	1	1	0	0.0
1	0	1	19	1.4
0	0	1	9	0.7
1	1	0	104	7.8
0	1	0	16	1.2
1	0	0	1170	87.6
*Total*			1336	
***On road crashes***				
1	1	1	18	2.4
0	1	1	0	0.0
1	0	1	19	2.5
0	0	1	9	1.2
1	1	0	72	9.5
0	1	0	11	1.5
1	0	0	626	82.9
*Total*			755	
***Collisions with a motor vehicle***				
1	1	1	18	15.0
0	1	1	0	0.0
1	0	1	17	14.2
0	0	1	7	5.8
1	1	0	7	5.8
0	1	0	3	2.5
1	0	0	68	56.7
*Total*			120	

### Completeness of the linked data

As no crashes identified in both the NMDS and CAS databases were found to be missing in the ACC database, the models containing both interaction terms ACC*NMDS and ACC*CAS were excluded. Table 4 shows model-based estimates and unconditional standard errors from the remaining six models. From these data, it was estimated that 477 crashes in general (95% CI: 362–629), 258 on-road crashes (95% CI: 197–338) and 24 collisions (95% CI: 17–32) were missing from all databases. That is, the completeness of the linked data was 73.7% (95% CI: 68.0–78.7%) for all crashes, 74.5% (95% CI: 69.1–79.3%) for on-road crashes, and 83.3% (95% CI: 78.9–87.6%) for collisions.

**Table 4:** Capture-recapture models estimating missing crashes

**Model**	**Variables included**	**Estimate**	**SE**	**AIC**	**AIC weight**	**Weighted estimate**	**Unconditional SE**
***All crashes***							
1	ACC NMDS CAS	5.23	0.22	84.87	0.00000003	0.00000014	0.00000001
2	ACC NMDS CAS ACC*NMDS	5.75	0.37	84.48	0.00000003	0.00000019	0.00000001
3	ACC NMDS CAS ACC*CAS	5.06	0.27	85.32	0.00000002	0.00000011	0.00000001
4	ACC NMDS CAS NMDS*CAS	5.33	0.22	54.87	0.09009773	0.48066236	0.03346642
5	ACC NMDS CAS ACC*NMDS NMDS*CAS	6.32	0.41	50.37	0.85614943	5.40872405	0.07995839
6	ACC NMDS CAS ACC*CAS NMDS*CAS	5.19	0.27	55.90	0.05375276	0.27913806	0.02753761
Model-averaged estimate						6.17	
Unconditional SE							0.14
***On-road crashes***							
1	ACC NMDS CAS	4.61	0.24	75.99	0.0000010926	0.0000050416	0.0000005142
2	ACC NMDS CAS ACC*NMDS	5.15	0.37	75.19	0.0000016262	0.0000083754	0.0000002457
3	ACC NMDS CAS ACC*CAS	4.38	0.32	76.40	0.0000008888	0.0000038942	0.0000006566
4	ACC NMDS CAS NMDS*CAS	4.74	0.25	53.42	0.0870396250	0.4128898692	0.0311739533
5	ACC NMDS CAS ACC*NMDS NMDS*CAS	5.69	0.41	48.83	0.8633010530	4.9139959238	0.0796703971
6	ACC NMDS CAS ACC*CAS NMDS*CAS	4.56	0.33	54.54	0.0496557143	0.2264598505	0.0271116407
Model-averaged estimate						5.55	
Unconditional SE							0.14
***Collisions with a motor vehicle***							
1	ACC NMDS CAS	2.45	0.36	60.47	0.0002105079	0.000514923	0.0000677993
2	ACC NMDS CAS ACC*NMDS	2.75	0.43	61.24	0.0001433835	0.000393918	0.0000255905
3	ACC NMDS CAS ACC*CAS	2.40	0.62	62.46	0.0000777519	0.000186721	0.0000374729
4	ACC NMDS CAS NMDS*CAS	2.78	0.37	45.81	0.3216029517	0.895471259	0.0453152851
5	ACC NMDS CAS ACC*NMDS NMDS*CAS	3.33	0.47	44.94	0.4970883194	1.656397698	0.0609100608
6	ACC NMDS CAS ACC*CAS NMDS*CAS	3.37	0.70	46.96	0.1808770856	0.609953708	0.0483519920
Model-averaged estimate						3.18	
Unconditional SE							0.15

SE, Standard Error.

### Agreement between the linked and self-reported data

There was a moderate agreement (kappa 0.55) between the linked and self-reported data for all crashes as well as crashes involving collisions, with the highest level of agreement observed with the claims data (Table 5). For 4.7% of participants who reported at least one crash (that required medical attention or reported to the police) in the preceding year, there was no crash record in the linked data. In contrast, in 5.6% of participants who did not report a crash, one or more crashes were recorded in the linked data. This disagreement was less pronounced for collisions.

**Table 5:** Agreement between linked and self-reported data

	**Agreement N (%)**		**Disagreement N (%)**		**% Total agreement**	**Kappa (95% CI)**	**Sensitivity (95% CI)**	**Specificity (95% CI)**	**PPV (95% CI)**	**NPV (95% CI)**
	**Both yes**	**Both no**	**SR yes LD no**	**SR no LD yes**						
***All crashes***										
At least one crash claimed to ACC	114 (7.8)	1201 (82.5)	59 (4.1)	82 (5.6)	90.3	0.56 (0.50, 0.63)	65.9 (58.3, 72.8)	93.6 (92.1, 94.9)	58.2 (50.9, 65.1)	95.3 (94.0, 96.4)
At least one crash requiring overnight hospital admission	10 (0.7)	1415 (97.8)	18 (1.3)	12 (0.2)	98.5	0.47 (0.28, 0.66)	34.5 (18.6, 54.3)	99.8 (99.3, 100.0)	76.9 (50.0, 93.8)	98.7 (97.9, 99.2)
**At least one crash requiring attention**	**118 (8.1)**	**1187 (81.5)**	**69 (4.7)**	**82 (5.6)**	**89.6**	**0.55 (0.49, 0.61)**	**63.1 (55.7, 69.9)**	**93.5 (92.0, 94.8)**	**59.0 (51.8, 65.8)**	**94.5 (93.1, 95.7)**
***Collisions with a motor vehicle***										
At least one collision claimed to ACC	20 (1.4)	1412 (97.0)	21 (1.4)	3 (0.2)	98.4	0.62 (0.48, 0.76)	48.8 (33.2, 64.6)	99.8 (99.3, 100.0)	87.0 (65.3, 96.6)	98.5 (97.7, 99.1)
At least one collision requiring overnight hospital admission	2 (0.1)	1443 (99.1)	8 (0.6)	3 (0.2)	99.3	0.26 (−0.03, 0.56)	20.0 (3.5, 55.8)	99.8 (99.3, 100.0)	40.0 (7.3, 83.0)	99.5 (99.0, 99.7)
At least one collision reported to police	10 (0.7)	1412 (97.0)	34 (2.3)	0 (0.0)	97.7	0.36 (0.20, 0.53)	22.7 (12.0, 38.2)	100.0 (99.7, 100.0)	100.0 (65.6, 100.0)	97.7 (96.7, 98.3)
**At least one collision requiring attention**	**22 (1.5)**	**1399 (96.1)**	**33 (2.3)**	**2 (0.1)**	**97.6**	**0.55 (0.41, 0.68)**	**40.0 (27.3, 54.1)**	**99.9 (99.4, 100.0)**	**91.7 (71.5, 98.5)**	**97.7 (96.7, 98.4)**

SR, Self-reports; LD, Linked Data.

When self-reports were considered as the gold standard, the linked data for all crashes had 63.1% sensitivity, 93.5% specificity, 59.0% positive predictive value (PPV) and 94.5% negative predictive value (NPV). The sensitivity was counter-intuitively lower but the specificity and predictive values were higher for collisions.

There were variations in agreement by participants’ demographic characteristics, amount of cycling, pre-existing health conditions and confidence in recalling crash events (Table 6). A higher level of agreement was associated with being younger, male and Māori, having a higher level of education, spending less time cycling, not having pre-existing medical conditions, being more socioeconomically deprived and having a higher degree of confidence regarding the accuracy of recall.

**Table 6:** Agreement between linked and self-reported data by participant characteristics

**Participants' characteristics**	**N**	**Agreement N (%)**		**Disagreement N (%)**		**% Total agreement**	**Kappa (95% CI)**
		**Both yes**	**Both no**	**SR yes LD no**	**SR no LD yes**		
***Age***							
16-35	158	16 (10.1)	125 (79.1)	5 (3.2)	12 (7.6)	89.2	0.59 (0.42, 0.77)
36-50	715	64 (9.0)	590 (82.5)	26 (3.6)	35 (4.9)	91.5	0.63 (0.54, 0.71)
51+	583	38 (6.5)	472 (81.0)	38 (6.5)	35 (6.0)	87.5	0.44 (0.33, 0.55)
***Gender***							
Male	1069	93 (8.7)	865 (80.9)	49 (4.6)	62 (5.8)	89.6	0.57 (0.49, 0.64)
Female	387	25 (6.5)	322 (83.2)	20 (5.2)	20 (5.2)	89.7	0.50 (0.36, 0.63)
***Ethnicity***							
Maori	46	3 (6.5)	40 (87.0)	1 (2.2)	2 (4.4)	93.5	0.63 (0.25, 1.00)
Non-Maori	1410	115 (8.2)	1147 (81.4)	68 (4.8)	80 (5.7)	89.5	0.55 (0.48, 0.61)
***Level of education***							
High school (secondary) or less	240	13 (5.4)	193 (80.4)	15 (6.3)	19 (7.9)	85.8	0.35 (0.18, 0.52)
Polytechnic	370	31 (8.4)	305 (82.4)	14 (3.8)	20 (5.4)	90.8	0.59 (0.47, 0.72)
University	846	74 (8.8)	689 (81.4)	40 (4.7)	43 (5.1)	90.2	0.58 (0.50, 0.66)
NZDep 2006 scores*							
1-3	752	61 (8.1)	600 (79.8)	43 (5.7)	48 (6.4)	87.9	0.50 (0.41, 0.59)
4-7	516	44 (8.5)	426 (82.6)	19 (3.7)	27 (5.2)	91.1	0.61 (0.50, 0.71)
8-10	174	13 (7.5)	149 (85.6)	6 (3.5)	6 (3.5)	93.1	0.65 (0.46, 0.83)
***Urbanity of residence***							
Main urban area	1165	98 (8.4)	943 (80.9)	58 (5.0)	66 (5.7)	89.4	0.55 (0.48, 0.62)
Others	277	20 (7.2)	232 (83.8)	10 (3.6)	15 (5.4)	91.0	0.56 (0.41, 0.72)
***Region of residence***							
Auckland	531	37 (7.0)	431 (81.2)	30 (5.7)	33 (6.2)	88.1	0.47 (0.36, 0.58)
Wellington	313	27 (8.6)	251 (80.2)	14 (4.5)	21 (6.7)	88.8	0.54 (0.41, 0.68)
Others	598	54 (9.0)	493 (82.4)	24 (4.0)	27 (4.5)	91.5	0.63 (0.54, 0.72)
***Pre-existing medical condition***^ƚ^							
Yes	296	23 (7.8)	237 (80.1)	21 (7.1)	15 (5.1)	87.8	0.49 (0.35, 0.63)
No	1160	95 (8.2)	950 (81.9)	48 (4.1)	67 (5.8)	90.1	0.57 (0.50, 0.64)
***Hours spent cycling a week***							
0-2	237	12 (5.0)	212 (89.5)	3 (1.3)	10 (4.2)	94.5	0.62 (0.43, 0.81)
3-5	552	47 (8.5)	444 (80.4)	31 (5.6)	30 (5.4)	88.9	0.54 (0.44, 0.64)
6+	667	59 (8.8)	531 (79.6)	35 (5.2)	42 (6.3)	88.5	0.54 (0.45, 0.63)
***Confidence of reporting having crashes***							
Sure or very sure	1413	117 (8.3)	1150 (81.4)	68 (4.8)	78 (5.5)	89.7	0.56 (0.49, 0.62)
Others	43	1 (2.3)	37 (86.1)	1 (2.3)	4 (9.3)	88.4	0.23 (−0.20, 0.68)

* 2006 New Zealand Deprivation Index with decile ten the most deprived neighbourhood and decile one the least.

ƚ Heart attack, stroke, cancer, diabetes or high blood pressure.

SR, Self-reports; LD, Linked Data.

## Discussion

### Main findings

Our findings revealed a substantial underestimation of bicycle crashes in administrative databases. The capture-recapture models estimated that the linked data were 73.7% complete for all crashes with negligible differences between on- and off-road crashes. The linked data were 83.3% complete for collisions. In comparison with self-reports, the linked data had 63.1% sensitivity, 93.5% specificity, and 59.0% PPV and 94.5% NPV for all crashes and 40.0% sensitivity, 99.9% specificity, 91.7% PPV and 97.7% NPV for collisions. Agreement between the linked and self-reported data varied across individual data sources and by participants’ demographic characteristics, amount of cycling, pre-existing medical conditions and recall confidence.

### Strengths and limitations

The bicycle crash data collected in this prospective cohort study were obtained through record linkage to four routinely collected databases. This resource efficient method of data collection was designed to minimise potential biases associated with loss to follow-up [32]. This also provided a unique opportunity to evaluate the completeness of bicycle crash records across the spectrum of severity. To the best of our knowledge, this is the first study to compare official vs. self-reported data on bicycle crashes. However, some limitations need attention.

In our capture-recapture analysis, all underlying assumptions may not be completely satisfied. First, the assumption that the study population is closed may be violated by death or emigration of some participants, thereby underestimating the findings [33]. However, such underestimation may not be substantial as only six deaths were identified from the Mortality Collection database and only 23 participants provided an overseas address at the follow-up survey. Moreover, ACC support is available to New Zealand residents if they return home with an injury sustained during an overseas trip of up to six months (or longer if they are travelling on business and paying income tax).

Second, the assumption that each individual has equal probability to be captured in each database may be violated if the probability differs by crash, personal, social and health service factors [21,34].

Third, the assumption that there are no lost marks between databases (mark integrity) may be violated if ascertainment of relevant cases is affected by inaccuracies in coding of bicycle crash data in each data source [22,25,35]. Miscoding may have resulted in failure to identify some bicycle crashes, thereby underestimating the capture-recapture counts [36]. This may account for the counter-intuitive finding of a lower sensitivity for collision crashes compared to all crashes. It is possible that some collisions were miscoded as ‘cyclist only’ crashes as observed previously in the UK [37]. Case ascertainment may also be affected by the quality of record linkage. Although the match rate by NHI was high (99%), mistakes may have occurred during extraction of bicycle crashes from each data source as a conservative approach was used to minimise false matches. While this served as a sensible strategy to estimate unbiased risk ratios in our subsequent analyses [38,39], it may have underestimated the capture-recapture counts [36].

In addition, the self-reported data, although used as the gold standard in this study, may not be accurate. Inaccuracies in recall or provision of socially desirable responses may have resulted in under- or over-reporting of bicycle crashes. Cyclists generally experience frequent minor crashes, which could make recall of crash experiences during a specified period difficult. In previous research, the injury rates were significantly underestimated if the recall periods were two months or more [19] and the ability to recall was influenced by number, type and severity of injuries, and time elapsed since the injury event [40,41]. Over-reporting, as observed in relation to motor vehicle crashes [42], is also likely as some reported crashes may have occurred prior to the specified recall period. Moreover, near misses or evasion crashes may have been reported as collisions with a motor vehicle. This may be another explanation for the counter-intuitively lower sensitivity for collisions compared to all crashes. While previous studies reported negative associations between self-reported motor vehicle crashes and social desirability scales [20,43], little is known about how this bias might impact self-reported bicycle crashes.

### Interpretation

Our findings extend the existing literature and inform future attempts to estimate the burden and risk of bicycle-related injuries. As in previous research [16,17], our findings show that at most 30% of self-reported bicycle crashes were attended by medical personnel or the police. Even in this category of crashes, traditionally used databases may not be complete. Overseas research mainly assessed the completeness of hospital and police databases with varying results [44-46]. A New Zealand study found that only 22% of hospital-reported bicycle crashes and 54% of those involving a collision with a motor vehicle appeared in police reports [14]. In this study, 13% of hospital reported crashes and 64% of collisions were linkable to police records whereas 39% of police reported crashes and 43% of collisions were linkable to hospital records.

Very few studies have estimated the completeness of combined databases. In a US study, hospital and police records, if combined, were 80% complete for automobile vs. child bicyclist collisions [44]. However, this level of completeness could be much lower if minor injuries were also considered. In this study, only 12% of bicycle crashes and 43% of collisions extracted from the linked data were recorded in hospital or police databases. To our knowledge, no other studies have assessed the completeness of individual or combined databases for relatively minor injuries.

Even though multiple data sources were used to capture a spectrum of injuries, our capture-recapture counts may still be underestimates given the limitations mentioned above. This is evident in comparisons with the self-reported data where the sensitivity of the linked data was lower than the completeness of data as estimated from the capture-recapture methods. If potential over-reporting is taken into account, however, the actual completeness of the linked data may lie between the two extremes, that is, between 63% and 74% for all crashes and between 40% and 83% for collisions.

In this study, agreement between the self-reported and official data was at most moderate although a higher level of agreement was observed in relation to motor vehicle crashes and unintentional injuries [47]. This may be because, compared to motor vehicle crashes, bicycle crashes occur more frequently and many are less severe, making them less likely to be recalled or coded properly. Our findings suggest that confidence ratings may be a useful tool in assessing the quality of recalled crash data as observed in previous research [29]. There were also variations in agreement by participants’ personal factors, in accordance with earlier research on motor vehicle crashes [48].

## Conclusions

There were underestimations and inaccuracies of bicycle crash data collected from different sources. This underscores the need to consider and account for potential biases due to outcome misclassification in our subsequent analyses as well as in other similar studies. Our findings also emphasise the need to improve the quality of individual data sources, to develop comprehensive record linkage techniques, and to enhance the validity and reliability of self-reported information so that all available data sources can be used reliably in our future attempts to capture a complete picture of important injuries.

## Abbreviations

ACC: Accident Compensation Corporation; CAS: Crash Analysis System; LD: Linked data; NHI: National Health Index; NMDS: National Minimum Dataset; NPV: Negative predictive value; PPV: Positive predictive value; SD: Standard deviation; SE: Standard error; SR: Self-reports.

## Competing interests

No competing interests including financial competing interests.

## Authors’ contributions

STT contributed to the conception and design of the study, acquisition, analysis and interpretation of data and drafting of the manuscript. AW and SA contributed to the conception and design of the study, interpretation of data and revision of the manuscript. All authors read and approved the final manuscript.

## Pre-publication history

The pre-publication history for this paper can be accessed here:

http://www.biomedcentral.com/1471-2458/13/420/prepub

## References

[B1] Andersen LB Schnohr P Schroll M Hein HO All-cause mortality associated with physical activity during leisure time, work, sports, and cycling to work Arch Intern Med 2000 160 11 1621 1628 10.1001/archinte.160.11.1621 10847255

[B2] Higgins PAT Exercise-based transportation reduces oil dependence, carbon emissions and obesity Environ Conserv 2005 32 03 197 202 10.1017/S037689290500247X

[B3] Bassett DR Jr Pucher J Buehler R Walking, cycling, and obesity rates in Europe, North America, and Australia J Phys Act Health 2008 5 795 814 19164816 10.1123/jpah.5.6.795

[B4] Oja P Titze S Bauman A de Geus B Krenn P Reger-Nash B Kohlberger T Health benefits of cycling: a systematic review Scand J Med Sci Sports 2011 21 4 496 509 10.1111/j.1600-0838.2011.01299.x 21496106

[B5] Tin Tin S Woodward A Thornley S Ameratunga S Cycling and walking to work in New Zealand, 1991–2006: regional and individual differences, and pointers to effective interventions Int J Behav Nutr Phys Act 2009 6 1 64 10.1186/1479-5868-1186-1164 19765318 PMC2754975

[B6] Ministry of Transport Comparing travel modes 2012 Wellington: Ministry of Transport

[B7] Sport New Zealand Sport and Recreation Profile: Cycling - Findings from the 2007/08 Active New Zealand Survey 2009 Wellington: Sport New Zealand

[B8] Kingham S Koorey G Taylor K Attracting the next 10% of cyclists with the right infrastructure New Zealand Cycling Conference: 12–13 November 2009; New Plymouth 2009

[B9] Mackie H 'I want to ride my bike': overcoming barriers to cycling to intermediate schools. New Zealand Transport Agency Research Report No. 380 2009 New Zealand Transport Agency: Wellington

[B10] Ministry of Transport Risk on the road. Pedestrians, cyclists and motorcyclists 2012 Wellington: Ministry of Transport

[B11] Tin Tin S Woodward A Ameratunga SN Injuries to pedal cyclists on New Zealand roads, 1988–2007 BMC Public Health 2010 10 655 610.1186/1471-2458-1110-1655 21034490 10.1186/1471-2458-10-655 PMC2989960

[B12] Munster D Koorey G Walton D Role of road features in cycle-only crashes in New Zealand. Transfund New Zealand Research Report No. 211 2001 Transfund New Zealand: Wellington

[B13] Elvik R Mysen AB Incomplete accident reporting: Meta-analysis of studies made in 13 countries Transp Res Rec 1999 1665 133 140 10.3141/1665-18

[B14] Langley JD Dow N Stephenson S Kypri K Missing cyclists Inj Prev 2003 9 4 376 379 10.1136/ip.9.4.376 14693904 PMC1731022

[B15] Tercero F Andersson R Measuring transport injuries in a developing country: an application of the capture–recapture method Accid Anal Prev 2004 36 1 13 20 10.1016/S0001-4575(02)00109-4 14572822

[B16] de Geus B Vandenbulcke G Int Panis L Thomas I Degraeuwe B Cumps E Aertsens J Torfs R Meeusen R A prospective cohort study on minor accidents involving commuter cyclists in Belgium Accid Anal Prev 2012 45 683 693 22269558 10.1016/j.aap.2011.09.045

[B17] Hoffman MR Lambert WE Peck EG Mayberry JC Bicycle commuter injury prevention: It is time to focus on the environment J Trauma 2010 69 5 1112 1119 10.1097/TA.0b013e3181f990a1 21068616

[B18] Tivesten E Jonsson S Jakobsson L Norin H Nonresponse analysis and adjustment in a mail survey on car accidents Accid Anal Prev 2012 48 401 415 22664706 10.1016/j.aap.2012.02.017

[B19] Jenkins P Earle-Richardson G Slingerland DT May J Time dependent memory decay Am J Ind Med 2002 41 2 98 101 10.1002/ajim.10035 11813214

[B20] af Wåhlberg AE Dorn L Kline T The effect of social desirability on self reported and recorded road traffic accidents Transp Res Part F Traffic Psychol Behav 2010 13 2 106 114 10.1016/j.trf.2009.11.004

[B21] Cryer C Langley J Developing indicators of injury incidence that can be used to monitor global, regional and local trends 2008 Dunedin: Injury Prevention Research Unit, University of Otago

[B22] Davie G Langley J Samaranayaka A Wetherspoon ME Accuracy of injury coding under ICD-10-AM for New Zealand public hospital discharges Inj Prev 2008 14 5 319 323 10.1136/ip.2007.017954 18836049

[B23] Thornley SJ Woodward A Langley JD Ameratunga SN Rodgers A Conspicuity and bicycle crashes: preliminary findings of the Taupo Bicycle Study Inj Prev 2008 14 1 11 18 10.1136/ip.2007.016675 18245309

[B24] Accident Compensation Corporation Annual Report 2012 2012 Wellington: ACC

[B25] Health Outcomes International Pty Ltd Methods and systems used to measure and monitor occupational disease and injury in New Zealand: NOHSAC Technical Report 2 2005 Wellington: National Occupational Health and Safety Advisory Committee (NOHSAC)

[B26] Ministry of Health National Minimum Dataset (Hospital Inpatient events): Data Mart—Data Dictionary V7.5 2012 Wellington: Ministry of Health

[B27] Ministry of Health Mortality Collection Data Dictionary Version 1.3 2009 Wellington: Ministry of Health

[B28] Davie G Samaranayaka A Langley JD Barson D Estimating person-based injury incidence: accuracy of an algorithm to identify readmissions from hospital discharge data Inj Prev 2011 17 5 338 342 10.1136/injuryprev-2011-040090 21795287

[B29] Cust A Armstrong B Smith B Chau J van der Ploeg H Bauman A Self-reported confidence in recall as a predictor of validity and repeatability of physical activity questionnaire data Epidemiology 2009 20 3 433 441 10.1097/EDE.0b013e3181931539 19106800

[B30] Cook LM Brower LP Croze HJ The accuracy of a population estimation from multiple recapture data J Anim Ecol 1967 36 1 57 60 10.2307/3014

[B31] Hook EB Regal RR Capture-recapture methods in Epidemiology: Methods and limitations Epidemiol Rev 1995 17 2 243 264 8654510 10.1093/oxfordjournals.epirev.a036192

[B32] Greenland S Response and follow-up bias in cohort studies Am J Epidemiol 1977 106 3 184 187 900117 10.1093/oxfordjournals.aje.a112451

[B33] Hook EB Regal RR Internal validity analysis: A method for adjusting capture-recapture estimates of prevalence Am J Epidemiol 1995 142 Supplement 9 S48 S52 10.1093/aje/142.Supplement_9.S48 7572987

[B34] Hauer E Hakkert A Extent and some implications of incomplete accident reporting Transp Res Rec 1988 1185 1 10

[B35] McDonald G Davie G Langley J Validity of police-reported information on injury severity for those hospitalized from motor vehicle traffic crashes Traffic Inj Prev 2009 10 2 184 190 10.1080/15389580802593699 19333832

[B36] Brenner H Effects of misdiagnoses on disease monitoring with capture-recapture methods J Clin Epidemiol 1996 49 11 1303 1307 10.1016/0895-4356(95)00026-7 8892499

[B37] Ward H Lyons RA Thoreau R Under-reporting of road casualties - Phase 1. Road Safety Research Report No. 69 June 2006 London: Department for Transport

[B38] Howe GR Use of computerized record linkage in cohort studies Epidemiol Rev 1998 20 1 112 121 10.1093/oxfordjournals.epirev.a017966 9762514

[B39] Blakely T Salmond C Probabilistic record linkage and a method to calculate the positive predictive value Int J Epidemiol 2002 31 6 1246 1252 10.1093/ije/31.6.1246 12540730

[B40] Langley J Cecchi J Williams S Recall of injury events by thirteen year olds Methods Inf Med 1989 28 1 24 27 2704299

[B41] Warner M Schenker N Heinen MA Fingerhut LA The effects of recall on reporting injury and poisoning episodes in the National Health Interview Survey Inj Prev 2005 11 5 282 287 10.1136/ip.2004.006965 16203836 PMC1730266

[B42] af Wåhlberg AE On the validity of self-reported traffic accident data Safety on Road International Conference SORIC'02: 2002; Manama, Bahrain 2002

[B43] Lajunen T Corry A Summala H Hartley L Impression management and Self-Deception in traffic behaviour inventories Pers Individ Dif 1997 22 3 341 353 10.1016/S0191-8869(96)00221-8

[B44] Dhillon PK Lightstone AS Peek-Asa C Kraus JF Assessment of hospital and police ascertainment of automobile versus childhood pedestrian and bicyclist collisions Accid Anal Prev 2001 33 4 529 537 10.1016/S0001-4575(00)00066-X 11426683

[B45] Cryer PC Westrup S Cook AC Ashwell V Bridger P Clarke C Investigation of bias after data linkage of hospital admissions data to police road traffic crash reports Inj Prev 2001 7 3 234 241 10.1136/ip.7.3.234 11565992 PMC1730741

[B46] Juhra C Wieskötter B Chu K Trost L Weiss U Messerschmidt M Malczyk A Heckwolf M Raschke M Bicycle accidents – Do we only see the tip of the iceberg?: A prospective multi-centre study in a large German city combining medical and police data Injury 2012 43 12 2026 2034 10.1016/j.injury.2011.10.016 22105099

[B47] Begg DJ Langley JD Williams SM Validity of self reported crashes and injuries in a longitudinal study of young adults Inj Prev 1999 5 2 142 144 10.1136/ip.5.2.142 10385836 PMC1730483

[B48] McGwin G Jr Owsley C Ball K Identifying crash involvement among older drivers: agreement between self-report and state records Accid Anal Prev 1998 30 6 781 791 10.1016/S0001-4575(98)00031-1 9805521

